# Mesenchymal stem cell-mediated Notch2 activation overcomes radiation-induced injury of the hematopoietic system

**DOI:** 10.1038/s41598-018-27666-w

**Published:** 2018-06-18

**Authors:** Areumnuri Kim, Sehwan Shim, Min-Jung Kim, Jae Kyung Myung, Sunhoo Park

**Affiliations:** 1 Laboratory of Radiation Exposure & Therapeutics, National Radiation Emergency Medical Center, Seoul, Korea; 2 Department of Pathology, Korea Institute of Radiological & Medical Science, Seoul, Korea

## Abstract

Radiation exposure severely damages the hematopoietic system. Although several radio-protectors have been proposed to prevent radiation-induced damage, most agents have limited efficacy. In the present study, we investigated whether mesenchymal stem cells (MSCs) could contribute to the expansion of hematopoietic cells and mitigate radiation-induced hematopoietic injury *in vitro* and *in vivo*. We found that co-culture with MSCs promoted hematopoietic progenitor/stem cell (HPSCs) maintenance by providing a bone marrow-like microenvironment. In addition, we showed that MSCs prevented radiation-induced damage to HPSCs, as evidenced by the lack of DNA damage and apoptosis. Intravenously injected MSCs rapidly migrated to the bone marrow (BM) and prevented loss of BM cellularity, which reduced lethality and ameliorated pancytopenia in the BM of whole body-irradiated mice. We demonstrated that MSC-derived Jagged1 attenuated radiation-induced cytotoxicity of HPSCs, and that this was mediated by Notch signaling and expression of downstream proteins Bcl2 and p63 in HPSCs. In addition, Notch2 depletion significantly reduced the MSC-mediated radio-protective effect in human- and mouse-derived HPSCs. Collectively, our data show that activation of Notch and its associated downstream signaling pathways prevent radiation-induced hematopoietic injury. Therefore, enhancing Jagged1-Notch2 signaling could provide therapeutic benefit by protecting the hematopoietic system against damage after radiation.

## Introduction

Hematopoietic progenitor/stem cells (HPSCs) are capable of self-renewal and lineage-specific differentiation into all blood cell types^1^. Human HPSCs exhibit a CD34+ and CD38− phenotype and are controlled by the bone marrow (BM) microenvironment^2^. The majority of HPSCs exist in a quiescent state in the BM, but can be activated by external stimuli to readily proliferate and differentiate, resulting in the loss of stem cell markers^3,4^.

Radiation exposure is cytotoxic to the hematopoietic system as evidenced by increased accumulation of DNA damage leading to induction of apoptosis. Moreover, radiation-induced injury of the hematopoietic system results in cytotoxicity and loss of stemness, especially of HPSCs^5,6^. Previous studies have suggested that various radio-protectors can improve the viability of hematopoietic cells^7–9^; however, most are only effective before radiation exposure, and do not offer further protective effects after irradiation. Thus, potential therapeutic approaches are needed to restore HPSCs lost due to irradiation.

The BM contains HPSCs and various stromal cells including mesenchymal stem cells (MSCs)^10^. MSCs are non-hematopoietic, multipotent stem cells that can differentiate into various tissue types such as cartilage, bone, muscle, and neuronal lineages^11^. MSCs are an important component of the BM niche, and function in bone development, and maintenance, expansion, and proliferation of hematopoietic lineage cells^12,13^. Therefore, many studies have reported their therapeutic effect against inflammation in preclinical studies using *in vivo* models^14–16^. Moreover, MSCs are thought to be ideal for cell-based therapy in regenerative medicine^17,18^. Based on these advantages, therapies using MSCs could be applied to treat radiation-induced damage. Although previous studies have shown that MSCs protect against radiation-induced damage of the BM, intestine, and brain, the therapeutic mechanism is not well known.

Notch signaling is a developmental pathway found in both embryonic and adult tissues^19^. In mammals, there are five Notch ligands (Delta-like [Dll] 1, 3, and 4 and Jagged 1 and 2) and four receptors (Notch 1–4). Notch is a transmembrane receptor that is cleaved to release its intracellular domain, which directly affects the transcription of target genes^20^. This proteolytic cleavage is activated by a ligand–receptor interaction that leads to cleavage by the ADAM and γ-secretase complex. This process plays a critical role in regulating hematopoiesis by mediating cell–cell communication^21,22^. In the hematopoietic system, Notch receptors that are expressed on HPSCs interact with ligands on BM stromal cells to modulate hematopoiesis and survival^23,24^. Activated Notch has been reported to play an important role in the regeneration of hematopoietic cells after radiation-induced BM injury, but the associated mechanism is still unclear.

In this study, we used human- and mouse-derived HPSCs to study the mechanisms by which MSCs regulate the prevention of radiation-induced damage to the hematopoietic system. We also explored the involvement of Notch signaling in the interaction between HPSCs and MSCs. Our findings suggest that treatment with MSCs might have therapeutic potential to restore the hematopoietic system of patients exposed to radiation.

## Materials and Methods

### MSCs and CD34+CD38− HSCs

Human umbilical cord blood (UCB) was obtained from the umbilical vein immediately after delivery, with the informed consent of the mother; the protocol was approved by the Boramae Hospital Institutional Review Board (IRB) and the Korea Institute of Radiological & Medical Science IRB (IRB No. K-1501-002-022). Mononuclear cells (MNCs) were isolated from UCB using Ficoll-Hypaque (Sigma, St. Louis, MO, USA) gradient centrifugation. Next, cells were sorted from the MNCs using a magnetic cell-sorting MACS CD34+CD38− isolation kit (Miltenyi Biotech, Auburn, CA, USA) following the manufacturer’s protocol. CD34+CD38− cells were cultured with StemMACS HSC expansion media containing HSC Expansion Cocktail (Miltenyi Biotech). Umbilical cord blood-derived MSCs were purchased from the ATCC (Manassas, VA) and cultured with MSC growth medium MSCGM (Lonza, Walkersville, MD, USA).

### Radiation exposure and *in vivo* MSC injection

Six-week-old male C57BL/6 mice were maintained under specific pathogen-free (SPF) conditions and were acclimated for at least 7 days before handling. The animals were exposed to whole body irradiation (IR) using an X-ray machine (X-RAD 320, N. Branford, CT, USA) at a dose rate 2 Gy/min. To analyze total blood cells, mice were exposed to IR (6 Gy) and then MSCs (1 × 10^6^ cell/mouse) were intravenously injected into the tail vein at 3 h post-IR. Peripheral blood samples were collected in 50 mM EDTA solution via lateral tail vein incision. Complete blood counts were performed with an automatic analyzer (Hemavet, Drew Scientific, Oxford, CT, USA). To determine the effect of MSCs on mouse survival, mice were irradiated with 6 Gy and then MSCs (1 × 10^6^ cells/mouse) or shJagged1-MSCs (1 × 10^6^ cells/mice) were injected into the tail vein at two time points (3 h and 3 days) after IR. To detect MSCs in the mouse BM, animals were exposed to IR (6 Gy) and then carboxyfluorescein diacetate N-succinimidyl ester (CFSE)-stained MSCs (1 × 10^6^ cells/mouse) were injected intravenously. Six days after IR, CFSE-MSCs was measured by flow cytometry and observed using a confocal laser scanning microscope (Leica, Bannockburn, IL, USA). All mouse experiments were performed in accordance with the Korea Institute of Radiological & Medical Science IACUC-approved protocol.

### Histology

Tibias were fixed in 4% paraformaldehyde at 4 °C for 3 days. After fixation, bones were decalcified and dehydrated in progressive concentrations of ethanol, cleared in xylene, and embedded in paraffin. The entire tibia was then sectioned longitudinally at 3 μm per section. To measure BM cell proliferation, sections from the center of the femur were stained with Ki67, Notch2, p63 (Abcam), and Bcl2 (Santa Cruz Biotechnology, Santa Cruz, CA, USA). Histologic staining was performed with hematoxylin and eosin.

### ELISA assay

Blood samples were obtained from rats at days 7 and 14 post-IR. Flt3 ligand was measured using a mouse/rat Flt3 ligand Quantikine ELISA kit (R&D Systems, Minneapolis, MN, USA) according to the manufacturer’s instructions. The optical density was measured using a microplate reader at 450 nm.

### Co-culture of CD34+CD38 HSCs with MSCs

Human MSCs were seeded and grown until 80% confluency in 6-well plates. HPSCs were exposed to ^137^Cs γ-rays using a Gamma Cell-3000 irradiator (MDS Nordion International, Ontario, Canada) at a dose rate of 5 Gy/min. MSC medium was removed and non-irradiated or irradiated CD34+CD38− cells (1 × 10^5^ cells per well) were added to 2 mL of HSC culture medium containing HSC Expansion Cocktail (Miltenyi Biotech). Cells were maintained at 37 °C in a humidified atmosphere containing 5% CO_2_. The co-cultures were incubated for 3 and 6 days, at which HSCs were harvested and analyzed.

### Apoptosis and FACS analysis

Measurement of apoptosis was performed as previously described^25^. To analyze cell surface marker expression in HPSCs, a FACSCanto was used (Becton & Dickinson, Franklin Lakes, NJ, USA). To calculate the frequency of HPSCs, human derived-CD34+CD38− cells were stained with anti-human antibodies against the lineage markers CD45, CD34, and CD38 (Miltenyi). Mouse BM lineage (Lin)-cKit+Sca+ cells were also assessed by flow cytometry. To analyze apoptosis, fresh human- and mouse-derived HPSCs were exposed to IR and treated with or without Jagged1. Recombinant human and mouse Jagged1 protein was purchased from Abcam (Cambridge, MA, USA). Human HPSCs were stained with Annexin V-FITC and propidium iodide and analyzed using FACS. Mouse HPSCs were stained with CD45-APC and Annexin V-FITC. Experiments were repeated three times.

### Immunofluorescence staining

Samples were prepared and stained as described previously^25^. Cells were exposed to radiation (2 or 4 Gy) for 24, 48, and 72 h. The primary antibodies used were specific for p-γH2AX (Invitrogen, Carlsbad, CA, USA), and DAPI (Sigma) was used for nuclear staining. Immunostained slides were observed using a confocal laser scanning microscope (Leica).

### Real-time PCR

Total cellular RNA was isolated using TRIzol reagent (Invitrogen) and subjected to reverse transcription to obtain total cDNA using Power SYBR Green Master Mix based qRT PCR assays (Thermo Fisher, Waltham, MA, USA). Real-time RT-PCR was performed with a LightCycler 489 Real-Time PCR System (Roche, Indianapolis, IN, USA) using the standard settings. Target gene expression was normalized to GAPDH. The sequences of the primers used are shown in Supplementary Table 1.

### Western blotting

Western blotting was performed as described previously^25^ using primary antibodies against the following proteins: cleaved PARP, Notch2, and Jagged1 (Cell Signaling Technology, Danvers, MA, USA); p63 (Abcam); and Bcl2 and β-actin (Santa Cruz Biotechnology).

### Bone marrow cell isolation and RNAi transfection assay

Mouse BM cells were harvested by crushing two tibias and two femurs from each mouse. For isolation of mouse hematopoietic progenitor cells, we used an EasySep magnet (Stemcell Technologies, Vancouver, BC) following the manufacturer’s protocol. Isolated cells were cultured with DMEM (Gibco) in the presence of murine SCF (100 ng/mL), IL-3 (10 ng/mL), and IL-6 (10 ng/mL). Cytokines were purchased from Miltenyi Biotech. BM cells (1 × 10^5^/well) were seeded into 6-well plates and transfected with 30 nM Notch2 siRNA (Dharmacon, Lafayette, CO, USA) or control non-targeting siRNA (Dharmacon) using the Magnetofection system, and 1.0 μg DNA and 1.0 μL of PolyMAG, according to the manufacturer’s protocol (Chemicell, Berlin, Germany). After 12 h, cells were exposed to IR (or left untreated) and then plated with MSCs for 24 h. A lentiviral shRNA Jagged1 was purchased from Sigma-Aldrich (St. Louis, MO). Transfection was performed using Oligofectamine (Life Technologies, Gaithersburg, MD), following the manufacturer’s protocol. Briefly, lentivirus was generated by transfecting 293 T cells with the shRNA vector. Viral particles were added to MSC and then cell cultures were selected with puromycin (2 μg/ml).

### Statistical analysis

Quantitative data are expressed as the mean ± standard deviation. Mean values were compared using one-way analysis of variance (ANOVA) and Student’s t-test. A P value < 0.05 was considered statistically significant. All experiments were repeated three times.

## Results

### UCB-derived MSCs block radiation-induced cell death of HPSCs

MSCs promote expansion and proliferation of hematopoietic cells. Thus, to confirm the effect of MSCs on hematopoietic cells, we performed co-culturing of HPSCs with BM-derived MSC (BM-MSC) or UCB-derived MSC (UCB-MSC). After 6 days, the co-cultures with MSCs had a slightly increased CD45+/CD34+ cells compared to the mono-cultured cells. In addition, the CD34+CD38− cells were significantly increased by co-culture with MSC compared with mono-cultured cells (Supplementary Figure 1A and B). Next, to determine whether BM-MSCs or UCB-MSCs mitigate radiation damage to hematopoietic cells, HPSCs were exposed to IR and then co-cultured with or without MSC. Six days after IR, the total number of CD34+CD38− cells had increased by nearly 2-fold in co-culture with UCB-MSC, compared to co-culture with BM-MSC (Supplementary Figure 2A). Moreover, analysis of apoptotic cell death revealed that UCB-MSC reduced IR-induced cell death by 80% to 32%, while BM-MSC decreased cell death by only 62% (Supplementary Figure 2B). These results showed that UCB-MSC might be better than BM-MSC as radio-protectors to rescue hematopoietic cells after IR-induced injury. Thus, we further studied the ability of UCB-MSC to inhibit radiation-induced damage to hematopoietic cells.

### MSC treatment rescues defective hematopoietic functions in radiation-damaged BM

**Figure 1:**
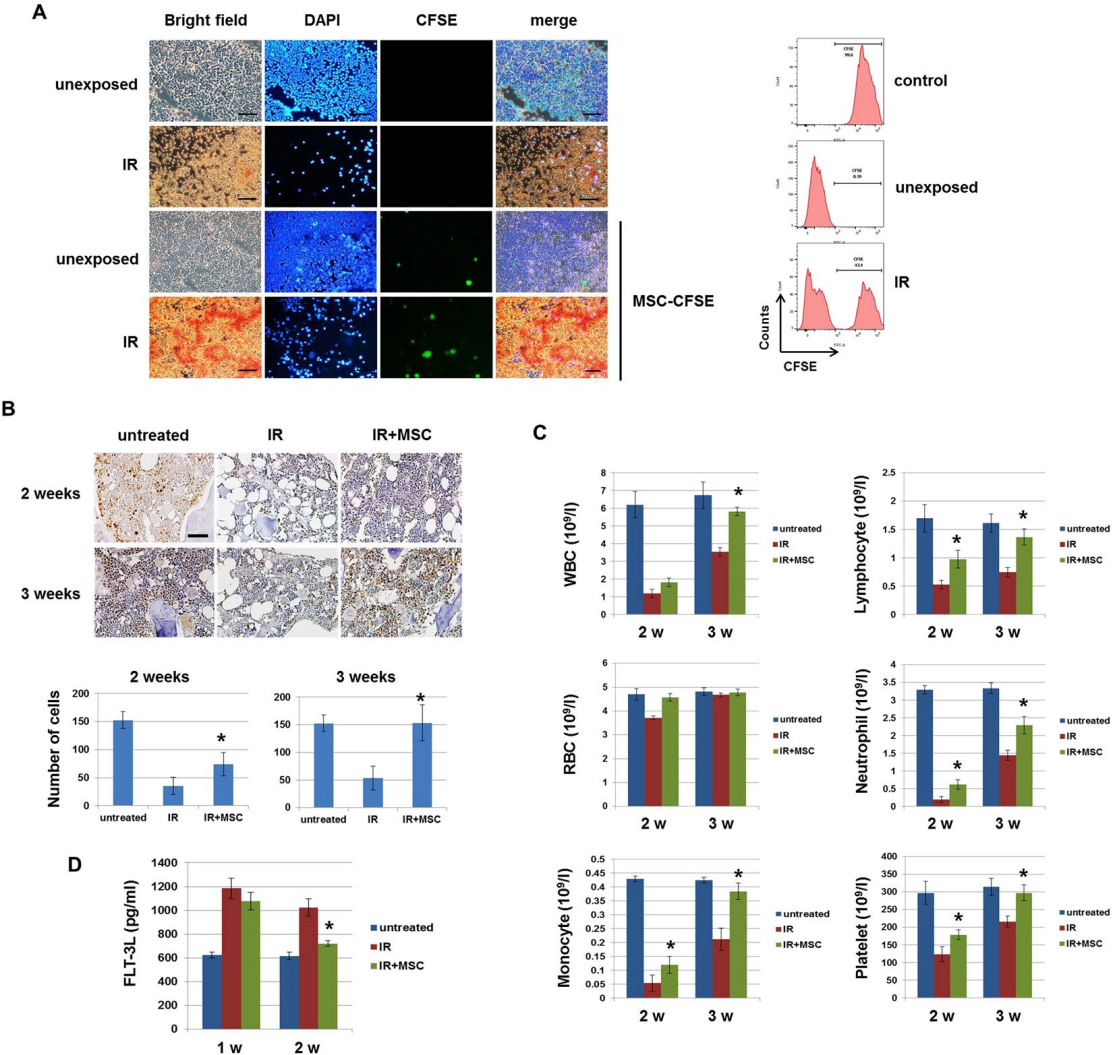
Mesenchymal stem cells (MSCs) protect against radiation-induced loss of bone marrow (BM) cellularity in mice. (**A**) Mice were injected with CFSE-stained MSC (1 × 10^6^ cells/mouse) via the tail vein after total body irradiation (IR; 6 Gy). Three days after IR, the BM of mice was isolated and CFSE-labeled MSCs were observed (left panel). CFSE expression was measured by flow cytometry (right panel). Bars indicate 100 μm. (**B**) Representative images of Ki-67 expression in the BM of mice at 2 and 3 weeks after IR. Average number of Ki67-positive cells are shown. Bars represent ± SD. *p < 0.05, for IR alone and IR + MSC. Bars indicate 100 μm. (**C**) Mice were subjected to IR following administration of MSCs and blood cell counts were assessed at the indicated times. Peripheral blood cell counts (total white blood cells (WBCs), red blood cells (RBCs), lymphocytes, platelets, neutrophils, and monocytes), showing the effects of MSCs, were observed on day 14 and 21 (n = 10). (**D**) Flt-3 ligand levels were measured by ELISA using the plasma of mice. All error bars indicate SEM. *p < 0.05.

To determine whether MSCs migrate to the BM *in vivo*, CFSE-labeled MSCs were injected into mice after IR. We found that MSC-CFSE migration was more efficient in the BM of radiation-exposed mice compared to unexposed mice (Fig. 1A). This result showed that radiation promoted the migration of injected MSCs to the damaged BM.

To investigate whether MSC treatment can overcome radiation-induced hematopoietic cell damage in the BM, we evaluated the effects of transplanted MSCs on BM function. We observed increased cell proliferation in the damaged BM after MSC transplantation compared to that in non-transplanted controls, as assessed by Ki-67 expression, at 2 and 3 weeks after IR (Fig. 1B). A defective hematopoietic system is known to result in pancytopenia, which is a reduction of circulating peripheral blood cells. We examined whether MSC administration could affect radiation-induced pancytopenia. Peripheral blood cell counts including total white blood cells, monocytes, lymphocytes, neutrophils, and platelets, were significantly elevated in the MSC-transplanted mice at 3 weeks post-IR (Fig. 1C). In contrast, the level of red blood cells was not changed by MSC administration, as evidenced by a slight decrease in the non-transplanted control group. Flt3 levels were found to be negatively correlated with the number of residual hematopoietic progenitors^26^. We found that expression of the Flt3 ligand was greatly increased in only the radiation-exposed mice, but it was rapidly decreased in MSC-transplanted animals at 2 weeks post-IR (Fig. 1D). Taken together, these data show that MSC treatment can enhance the hematopoietic function of the radiation-damaged BM by protecting against pancytopenia.

### MSCs suppress loss of stemness and apoptosis in radiation-damaged HPSCs

**Figure 2:**
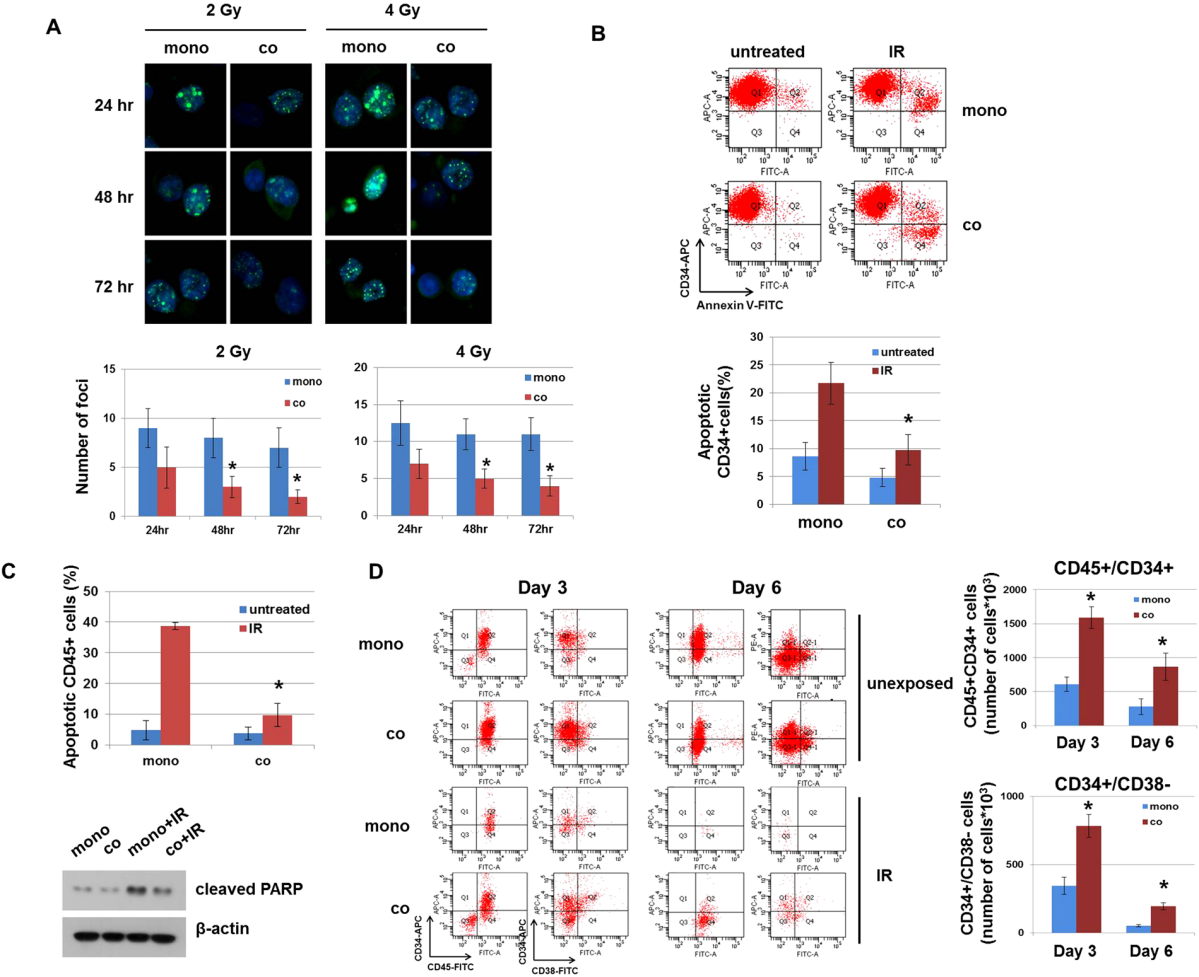
MSCs prevent IR-induced cytotoxicity of hematopoietic progenitor/stem cells (HPSCs). HPSCs were exposed to IR and cultured with/without MSCs. (**A**) Immunofluorescence staining for phospho γH2AX in HSCs 24, 48, and 72 h after IR (2 or 4 Gy) exposure. A total of 1000 cells were evaluated for hematopoietic cells. Graphs show the number of γH2AX foci in HPSCs. DAPI was used for nuclear staining. *p < 0.05, for mono-culture and co-culture. (**B**) Apoptotic cell death of HPSCs was analyzed with CD34-APC and Annexin V-FITC at day 3 after IR. *p < 0.05, for mono-culture and co-culture. (**C**) Whole cells stained with CD45 and Annexin V at day 3 after IR. Apoptotic cells (CD45+Annexin V+) were analyzed by flow cytometry. Whole cell lysates were used to assess cleaved PARP by western blotting of IR-exposed hematopoietic cells at day 3. β-actin was used as a protein loading control. *p < 0.05, for mono-culture and co-culture. (**D**) On days 3 and 6 following IR (4 Gy), cells were evaluated for CD45-FITC/CD34-APC and CD38-FITC/CD34-APTC expression using flow cytometry (left panel). Graphs show the number of CD45+CD34+ cells and CD34+CD38− cells (right panel). *p < 0.05, for mono-culture and co-culture.

Because MSCs have the ability to improve BM recovery after radiation injury^27^, we investigated the mechanism by which this occurs. γH2AX, as a marker of DNA damage, is a useful biomarker to assess the effects of exposure to IR^28^. We found that the number of γH2AX foci was significantly decreased in co-cultured HPSCs, compared to mono-cultured cells (Fig. 2A). Next, we examined whether DNA damage promotes apoptosis in HPSCs; for this, HPSCs were exposed to IR and cultured with or without MSCs. Radiation exposure increased apoptosis in mono-cultured CD34+ cells by 22.8%, in contrast to only 9.8% in co-cultured CD34+ cells (Fig. 2B). In addition, upon co-culture with MSCs, apoptotic CD45+ cells were significantly decreased by nearly 4-fold compared to mono-cultured cells. We also observed that cleaved PARP, a marker of apoptosis, was decreased in co-cultured HPSCs (Fig. 2C). Thus, these results show that co-culture with MSCs attenuates radiation-induced cytotoxicity of HPSCs. To determine whether IR affects HPSC populations, hematopoietic cell markers were analyzed in mono- or co-cultured HPSCs after IR. CD34+CD38− cells were significantly increased, by approximately 2-fold, in the presence of MSCs, as compared to HPSCs alone. In addition, co-cultured HPSCs exhibited up-regulation of CD34+ expression by approximately 3-fold, compared to mono-cultured HPSCs (Fig. 2D). Therefore, MSCs protect HPSCs from radiation-induced loss of stemness, which leads to differentiation of hematopoietic lineage cells.

### Activated Notch signaling protects hematopoietic cells from radiation-induced cytotoxicity

**Figure 3:**
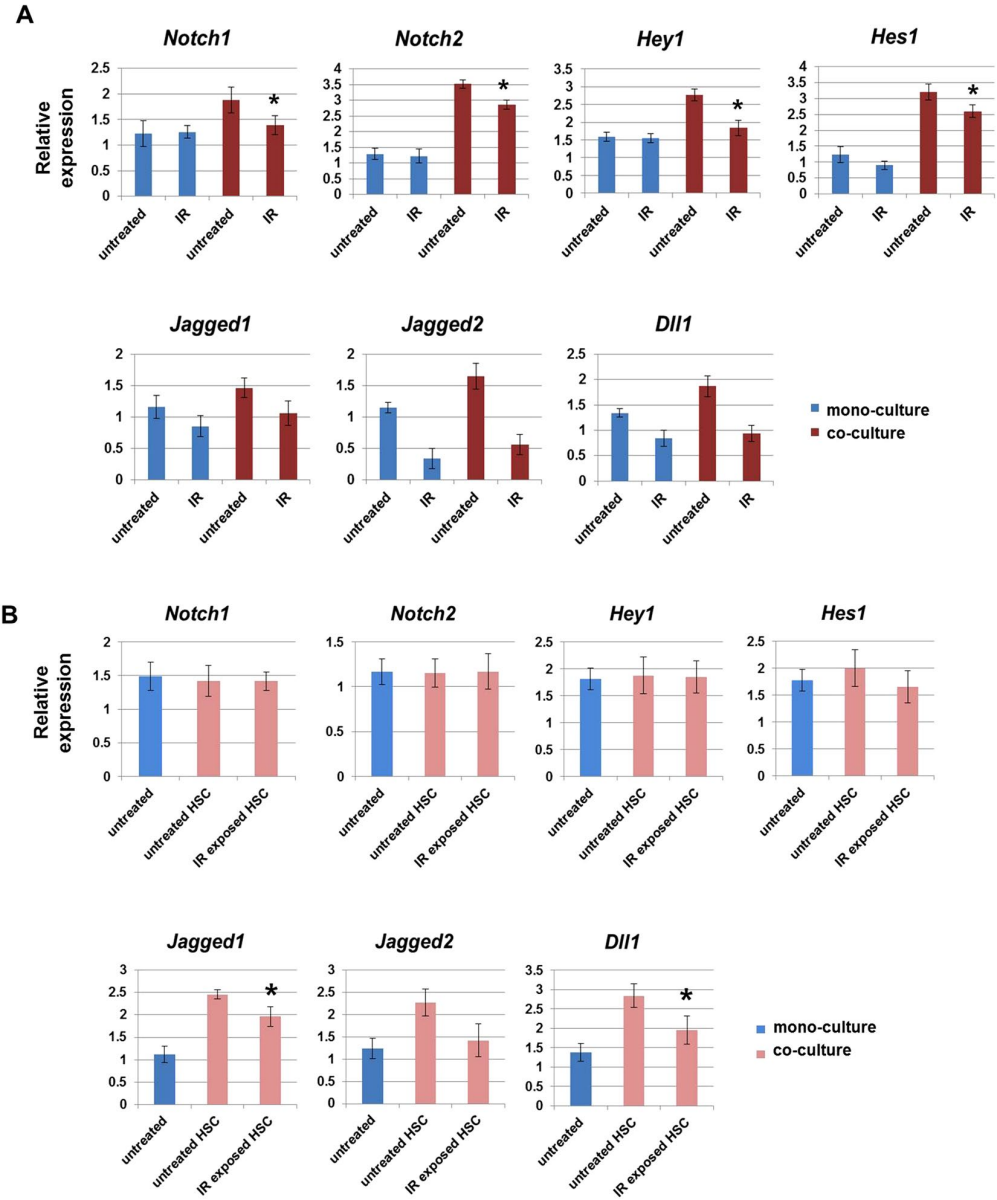
Activated Notch signaling protects against IR-induced damage to HPSCs. HPSCs were subjected to IR (4 Gy) and cultured with/without MSCs. At day 3 post-IR, the expression levels of *Notch1*, *Notch2*, *Hey1*, *Hes1*, *Jagged1*, *Jagged2*, *and Dll1* were analyzed in HPSCs (**A**) or MSCs (**B**) using qPCR. *p < 0.05, for mono-culture and co-culture.

Notch-mediated cell-to-cell signaling is involved in differentiation and homeostasis of BM stromal cells and hematopoietic cells^19^. Thus, we expected that Notch signaling would be associated with the radio-protective effect of MSCs in hematopoietic cells. To determine whether interactions between HPSCs and MSCs activate Notch signaling, we analyzed the expression of Notch-associated genes such as *Notch1*, *Notch2*, *Hey1*, *Hes1 Jagged1*, *Jagged2*, and *Dll1* in HPSCs and MSCs. The expression of *Notch2* and *Hes1* were greatly enhanced, by 3-fold, in co-cultured HPSCs compared to mono-cultured HPSCs. Elevated Notch2 expression also remained high after IR. In addition, the expression of *Notch1* and *Hey1* was increased by 2–2.5-fold upon HPSC co-culture, but levels of these markers were decreased following radiation exposure (Fig. 3A). Next, we observed that the Notch ligands *Jagged1* and *Dll1* were significantly up-regulated in MSCs co-cultured with HPSCs (Fig. 3B). To show that activated Notch is involved in the inhibition of apoptosis in HPSCs, the γ-secretase inhibitor DAPT was used to treat mono- and co-cultured cells. As expected, radiation-induced apoptosis was attenuated in HPSCs co-cultured with MSCs compared to mono-cultured cells, whereas DAPT treatment overrode the protective effects of MSC and HPSC co-culture (Supplementary Figure 4). Moreover, the combined injection of MSCs and DAPT into mice resulted in a greater loss of BM cellularity and higher mortality compared to IR alone (Supplementary Figure 5). These results indicate that treatment with DAPT impedes the regenerative effect of MSCs, thereby promoting radiation-induced multi-organ failure. Thus, MSCs prevented IR-induced cytotoxicity to the hematopoietic system, which implies that the activation of Notch signaling is involved in this process.

### Activated Notch signaling suppresses BM damage through inhibition of p63 expression

**Figure 4:**
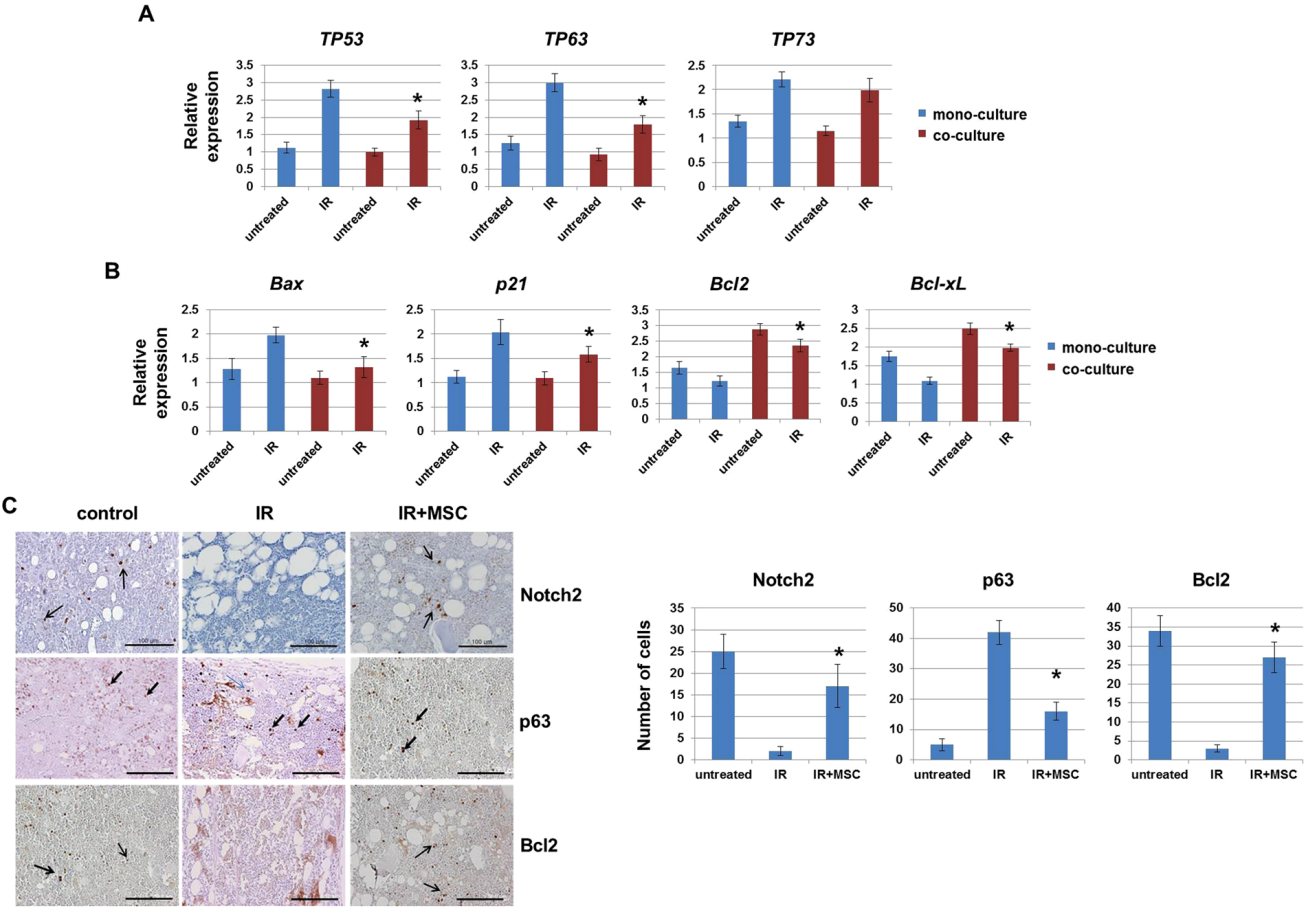
Co-culture with MSCs regulates Notch2 and associated p53 family member proteins. HPSCs were exposed to IR (4 Gy) and incubated with/without MSCs for 3 days. Expression levels of *TP53*, *TP63*, *TP73* (**A**), *Bax*, *p21*, *Bcl2*, and *Bcl-xL* (**B**) were assessed by qPCR. (**C**) Mice were treated with MSCs (1 × 10^6^ cells/mouse) after total body IR (6 Gy). Two weeks after IR, Notch2, p63, and Bcl2 expression were detected in mouse BM using immunohistochemical staining (left panel). Bars indicate 100 μm. Average numbers of Notch2, p63, and Bcl2-positive cells are shown (right panel). Bars represent ± SD. *p < 0.05, for IR alone and IR + MSC.

We next investigated which molecules were involved in the protective effect of Notch signaling against IR-induced cytotoxicity. p53 family members p63 and p73 are important in the cellular response to DNA damage^29^, and previous studies have shown that Notch signaling regulates p63 expression^30^. Thus, we determined whether activated Notch suppressed DNA damage through p53 family members in mono- or co-cultured HPSCs. As shown in Fig. 4A, radiation exposure increased p53, p63, and p73 expression by approximately 2.5-fold in mono-cultured cells compared to untreated cells. However, co-culture with MSCs significantly reduced *TP53* and *TP63* expression in HPSCs. In addition, co-cultured cells showed down-regulation of p53 associated with Bax and p21 expression, with increased expression of the anti-apoptotic genes Bcl2 and Bcl-xL (Fig. 4B). We also analyzed whether the protein expression of Notch2, p63, and Bcl2 was altered by IR and MSCs in mouse BM. IR resulted mostly in decreased Notch2 and Bcl2; in contrast, p63 expression was decreased in the MSC-injected group, which was similar to the *in vitro* results (Fig. 4C). Using immunofluorescence staining, we confirmed that co-localization of Notch2 and Bcl2 was up-regulated by MSC injection. IR-induced p63 expression was also decreased in MSC-injected BM of mouse (Supplementary Figure 6). Thus, MSCs enhance Notch2 expression on hematopoietic cells, which overcomes IR-induced cytotoxicity through inhibition of p63 expression.

### Interaction between Jagged1 and Notch2 blocks IR-induced apoptosis of HPSCs

**Figure 5:**
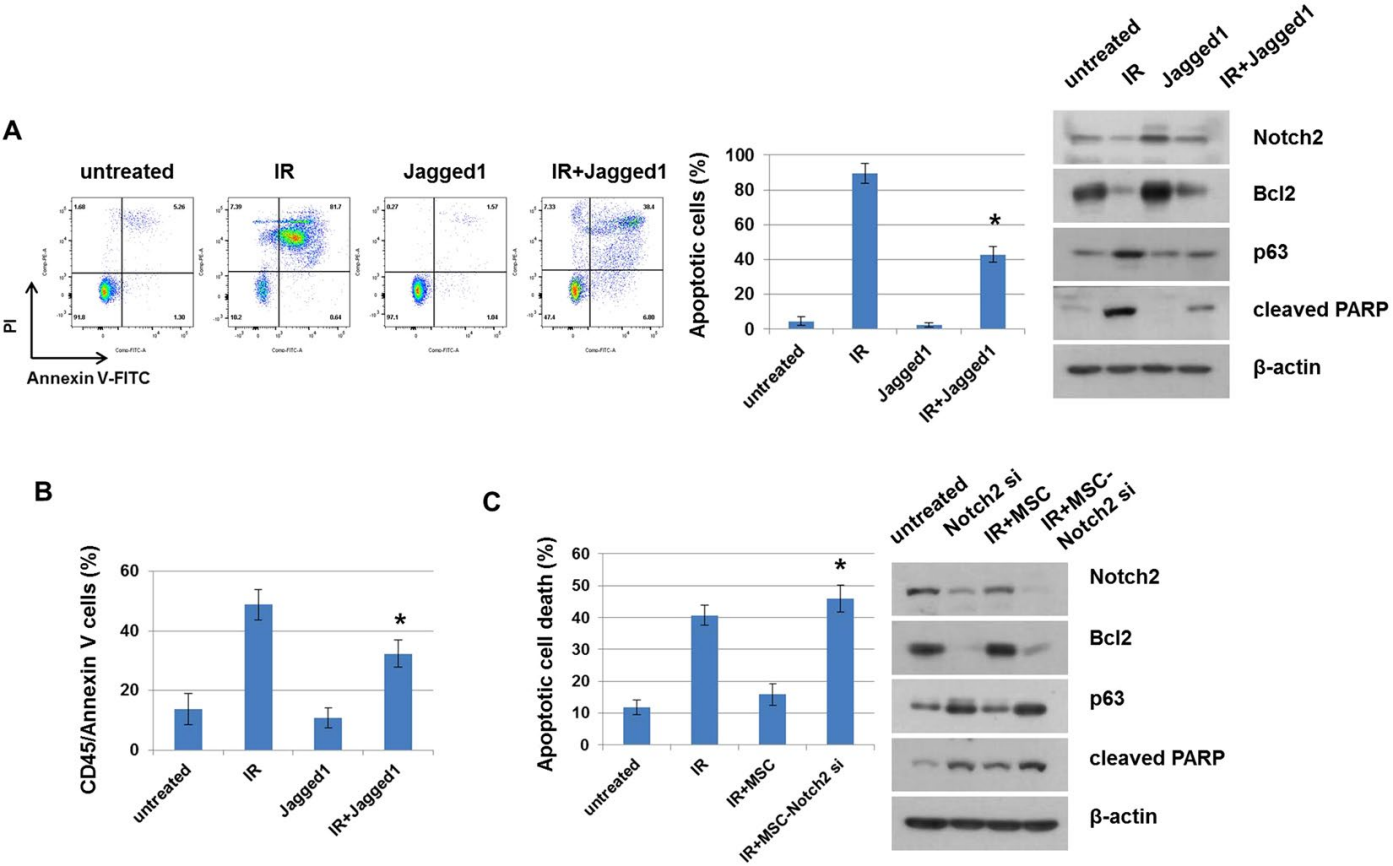
Treatment with Jagged1 protects against cytotoxicity of HPSCs. (**A**) HPSCs were exposed by IR (4 Gy) and then treated with or without Jagged1 for 3 days. Apoptotic cells (Annexin V and PI-positive) were measured by flow cytometry. *p < 0.05, for IR alone and IR + Jagged1 (left panel). The expression of Notch2, Bcl2, p63, and cleaved PARP were detected by western blotting. β-actin was used as a protein loading control (right panel). (**B**) IR-exposed mouse BM cells were incubated with Jagged1 for 3 days. Apoptotic cells (CD45 and Annexin V-positive) were measured by flow cytometry. *p < 0.05, for IR alone and IR + Jagged1. (**C**) HPSCs were transfected for 24 h with Notch2 siRNA, which was followed by IR exposure of MSCs for an additional 24 h. Cells were stained with CD45/Annexin V and apoptotic cells were analyzed by flow cytometry. *p < 0.05, for IR + MSC and IR + MSC-Notch2 siRNA (left panel). Notch2, Bcl2, p63, and cleaved PARP levels were analyzed by western blotting. β-actin was used as a protein loading control (right panel).

To determine whether the Notch ligand–receptor interaction plays an important role in HPSC differentiation, we used the Notch ligand Jagged1. We found that treatment with Jagged1 prevented the loss of CD34+CD38− cells, as compared to untreated cells (Supplementary Figure 7). This result indicates that Jagged1 attenuates the differentiation of HPSCs. In addition, Jagged1 treatment reduced IR-induced apoptosis from 84% to 41% in human HPSCs (Fig. 5A). This apoptotic rate is similar to the effect observed when we evaluated the radio-protection effect of MSCs. We also confirmed that Jagged1 increased expression of Notch2 and its associated proteins Bcl2 and p63 at the protein level, but decreased levels of cleaved PARP. In mouse HPSCs, Jagged1 also prevented radiation-induced apoptosis of CD45 cells by 52% to 33% (Fig. 5B). However, Dll1 did not significantly protect against radiation-induced cell death in human and mouse HPSCs (data not shown). Next, we depleted Notch2 using specific siRNAs to determine whether it is required for the prevention of IR damage to hematopoietic cells. Notch2 depletion significantly increased apoptosis in co-cultured HPSCs following IR. Additionally, Notch2 depletion decreased expression of Bcl2 family proteins but increased p63 and cleaved PARP in co-cultured HPSCs (Fig. 5C). Therefore, MSC-derived Jagged1 can activate Notch2 in HPSCs, which can function to reduce radiation-induced cytotoxicity to HPCs.

### Knockdown of Jagged1 in MSC impedes recovery from radiation-induced BM injury

**Figure 6:**
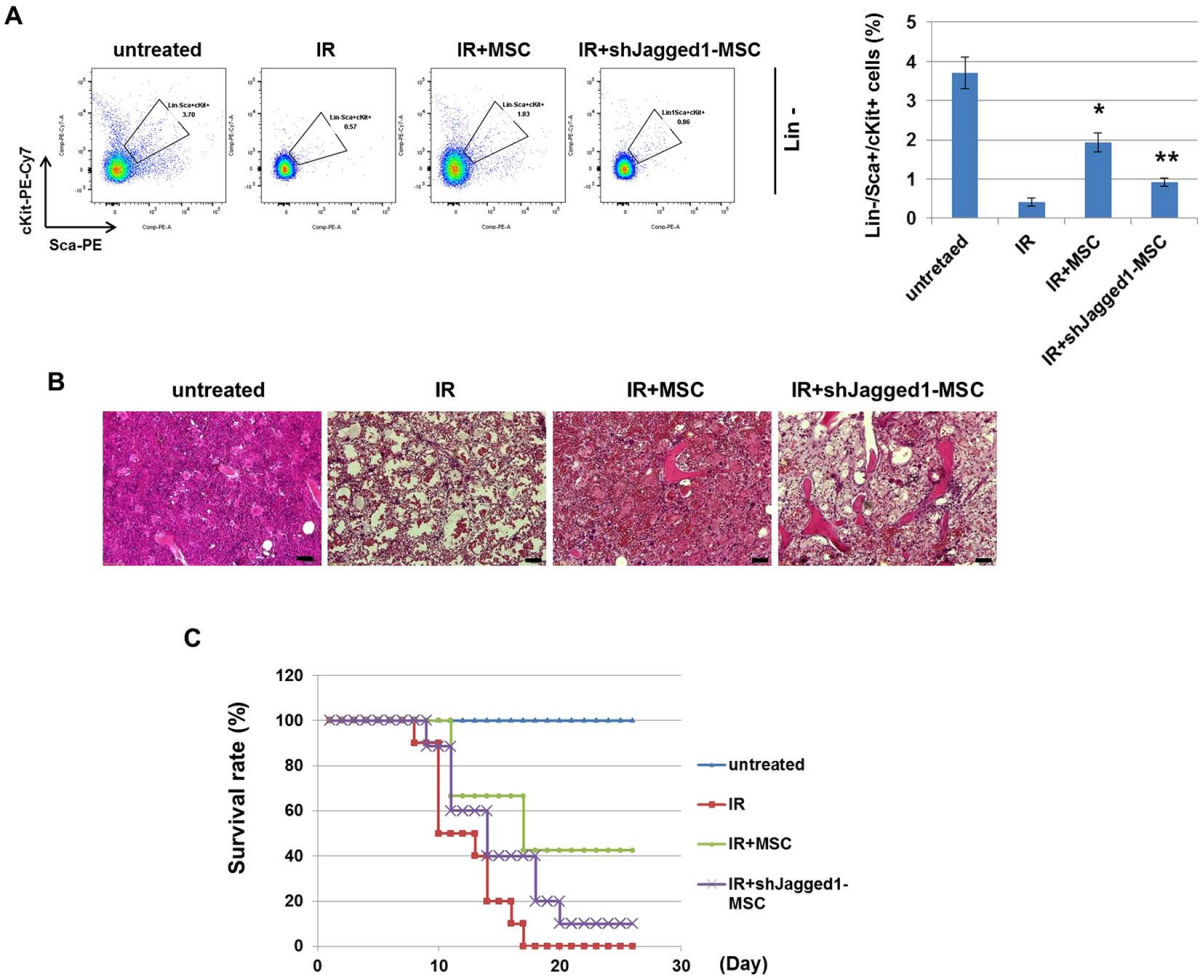
Depletion of Jagged1 in MSCs could not mitigate IR-induced loss of BM cellularity and lethality in mice. Mice were exposed to IR (6.5 Gy) followed by tail vein injection of MSCs (1 × 10^6^ cells/mouse) or shJagged1-MSCs (1 × 10^6^ cells/mouse). (**A**) On day 3 after IR, mouse BM was stained with Lin/Sca/cKit to detect mouse hematopoietic stem cells by flow cytometry. Representative images show Lin−/Sca+/cKit+ cells. *p < 0.05, for IR alone and IR + MSC; **p < 0.05, for IR + MSC and IR+ shJgged1-MSC. (**B**) Representative images are shown for H&E staining of mouse femurs at 7 days after IR. Scale bar = 50 μm. (**C**) Mice were observed for survival over 25 days (n = 10). The graph shows survival curves.

Our results show that MSC-induced Jagged1 can reduce radiation-induced cytotoxicity of hematopoietic cells *in vitro*. To determine whether knockdown of Jagged1 plays a major role in IR-induced BM damage, we used shRNA to deplete Jagged1 in MSC and injected MSCs or shJagged1-MSCs into mice after IR. MSC treatment resulted in restoration of mouse HPSCs (Lin−Sca+cKit+) to 1.8–2.5%, but shJagged1-MSC could not prevent radiation-induced depletion of most HSCs in the mice (Fig. 6A). Moreover, IR-exposed mice treated with MSCs showed restoration of the BM cellular content, whereas shJagged1-MSCs could not protect against loss of BM cellularity (Fig. 6B). We next investigated whether knockdown of Jagged1 in MSCs affected the survival of mice after IR. Mice received a total body irradiation dose of 6.5 Gy, which is a lethal radiation dose with 0% survival. As shown in Fig. 6C, all mice died in the IR-exposed group on day 16 post-IR. However, 43% of mice treated with MSCs while only 10% of mice treated with shJagged1-MSCs survived to 25 days after IR. This result indicates that Jagged1 majorly regulates hematopoietic damage by IR, but other MSC-induced factors may be involved. Taken together, these results indicate that Jagged1 expression by MSCs can protect against radiation-induced lethality, which suggests that MSC-based treatment could have potential therapeutic value for acute radiation syndrome.

## Discussion

In the current study, we demonstrate that activated Jagged1-Notch2 signaling can overcome radiation-induced injury to hematopoietic cells through inhibition of p63 *in vitro* and *in vivo*. We found that radiation exposure resulted in severe disruption of HPSCs in the BM, thereby increasing cellular lethality. Additionally, expression of Jagged1 by MSCs enhanced expression of Notch receptors on HPSCs, leading to protection against radiation-induced cytotoxicity. Our results indicate that Notch2 activation contributes to restoration of the BM, which suggests that MSC treatment could be an attractive therapeutic strategy for patients with radiation exposure.

The main result of radiation damage is impairment of HPSCs and their associated compartments^31,32^. Thus, HPSC recovery could be important to ameliorate radiation-induced injury. Several chemicals or compounds have been suggested to promote recovery from radiation-induced damage to the BM^7–9^, but most exhibit toxicity and insufficient radio-protective effects^33^. In addition, limited therapeutic options are currently available for radiation exposure such as that resulting from radiation therapy or nuclear accidents^34^. Two granulocyte colony-stimulating factor (G-CSF)-based growth factors, Neupogen and Neulasta, have recently been approved by the FDA as radio-mitigators for patients undergoing radiation therapy for cancer^35^. However, to date, there are no FDA-approved radio-protectors for acute radiation syndrome.

A previous study by Jing *et al*. reported that co-culture with MSCs increased the expansion and maintenance of hematopoietic stem cells^36^. Specifically, three-dimensional culture of MSCs was critical to mimic the physiological microenvironment *ex vivo*^37,38^. Based on *in vitro* data, current clinical studies have proposed that MSC-based therapies are safe and have the potential to effectively treat hematopoietic diseases. However, knowledge regarding the mechanism of action underlying the therapeutic efficacy of MSCs is lacking. Therefore, in this study, we investigated whether treatment with MSCs could contribute to suppression of radiation-induced BM damage, and the mechanisms associated with radio-protection.

MSCs are found in and isolated from various tissues such as the BM, heart, UCB, and adipose^39^. In early studies, BM-derived MSCs were shown to be effective in a variety diseases, but recently UCB-MSCs were considered most suitable because of the similarity to BM-MSC, and their several advantages, including lower immunogenicity, higher *in vitro* expansion, noninvasive collection, and differentiation potential^40^. We also observed that both co-cultured with BM-MSC and UCB-MSC resulted in high proportions of CD34+ CD38− HPSCs, compared to mono-cultured cells (Supplementary Figure 1). These results indicate that MSCs contribute to HPSC maintenance, which suggests that they could be applied to the expansion of hematopoietic cells *in vitro*.

Notch is a transmembrane receptor that regulates important cellular signaling events such as proliferation, differentiation, apoptosis, and tissue renewal through cell–cell communication^41^. Notch receptors and ligands are widely expressed in the hematopoietic system, indicating that Notch has an important function in hematopoiesis^42^. Interaction of Notch receptors with ligands leads to cleavage and release of the Notch intracellular domain, which translocates to the nucleus and functions as a transcriptional activator. HES and HEY genes encoding basic helix-loop-helix (bHLH) transcriptional regulators are target genes modulated by this mechanism^43^. In the hematopoietic system, *Hes1* plays a major function among the Notch target genes in BM of HSCs differentiation^44^. We also observed that *Hes1* are significantly increased in HPSCs co-cultured with both UCB-MSC and BM-MSC compared with mono-cultured cells. However, assessing the apoptosis rate and cell viability revealed that co-culture of HPSCs with UCB-MSCs mitigated the effects of IR better than co-culture with BM-MSC (Supplementary Figure 2). A previous study reported that senescence was achieved by UCB-MSCs more slowly than BM-MSCs, which indicates that p53 and p21 expression are involved^45^. In addition, we found that the expression of *Hes1* was higher in HPSCs co-cultured with UCB-MSCs than co-cultured with BM-MSCs after IR (Supplementary Figure 9). Based on this study, we expected that BM-MSC could not prevent cytotoxicity of HPSCs from radiation because this cell population is more sensitive to radiation-induced senescence than UCB-MSC. Thus, we further investigated the mechanism of the radio-protective effect in the hematopoietic system using co-culture with UCB-MSC.

Previous studies have shown that Notch activation during *ex-vivo* expansion of HPSCs might be directly correlated with the capacity to regulate cell cycle and hematopoietic recovery under conditions of stress^46^. Moreover, recent studies reported that different Notch ligands affect specificity by interacting with Notch receptors; however, distinct functions for each ligand in many tissues are still unknown^47,48^. We found that *Notch1*, *Notch2*, *Hes1 and Hey1* expression are significantly up-regulated compared to mono-cultured cells after radiation exposure. We also demonstrated that up-regulation of *Jagged1* and *Dll1* in MSCs can activate Notch2 on HPSCs, resulting in up-regulation of CD34 in CD34+CD38− cells and inhibition of cell division. A previous study reported that treatment with the soluble Notch ligand Dll1 mitigated IR-induced hematopoietic damage through colony-stimulating factor 2 receptor beta 2^49^. However, we did not observe radio-protective effects in Dll1-treated HPSCs (data not shown). A previous study by Radtke *et al*. demonstrated that Jagged1-mediated Notch signaling was dispensable for HSC self-renewal and differentiation^46^. Lu and colleagues showed that endothelial cell-secreted Jagged1 and its binding to receptors activated Notch signaling^50^. Moreover, the Notch2-Jagged1 interaction has been reported to regulate erythropoiesis through stem cell factor signaling^51^. We also found that treatment with Jagged1 activates Notch2 and overcomes radiation-induced DNA damage and apoptosis in hematopoietic cells. We confirmed that knockdown of Notch2 using siRNA increased radiation-induced cytotoxicity in hematopoietic cells.

Recent studies have shown that Fringe glycosyltransferases are associated with Notch signaling, regulating the interaction between receptors and ligands^52^. In mammalian, three Fringe family genes have been identified as Lunatic Fringe (Lfng), Manic Fringe (Mfng) and Radical Fringe (Rfng)^52^. All Fringe genes are involved in the differentiation of T and B cells. Although IR exposure greatly reduced Fringe genes expression in HPSCs, expression of Lfng and Mfng were restored by Jagged1 treatment compared to Dll1 treatment (Supplementary Figure 10). A previous study by Singh *et al*. reported that Jagged1 and Mfng play important role in regulating Notch function during hematopoietic development^53^. Taken together, MSC-derived Jagged1 can significantly promote Notch2 signaling, which is not only important for HPSC maintenance, but also for mitigating radiation-induced damage.

DNA damage is a major cause of radiation-induced injury of the hematopoietic system. Activated p53 promotes DNA damage and cell death, which is dependent on the up-regulation of p63 and p73^29^. The structural organization of p63 and p73 is similar to p53, however, each family member is also controlled by some specific genes^54,55^. A previous study by Horsley *et al*. revealed that repression of Notch signaling promoted p63^30^. In addition, the interaction between Notch and p63 regulates cancer growth^56^ and epidermal differentiation^57^. In our results, p53 family members were significantly increased by IR, but co-culture with MSC significantly reduced p63 expression *in vitro* and *in vivo* (Fig. 4). We also confirmed that treatment with Jagged1 decreased p63 expression by IR, whereas knockdown of Notch2 increased p63 in HPSCs under co-culture conditions.

The interaction of Bcl2 and the p53 gene regulates apoptosis. Chen *et al*. reported that Notch ligand increased the phosphorylation of STAT3 and Erk1/2 and up-regulated the expression of Bcl2^49^. The switch from survival to apoptosis is regulated by Bcl2 family proteins in irradiated HSCs^28^. We found that expression of the anti-apoptotic Bcl2 family genes including Bcl2 and Bcl-xL was increased in co-cultured HPSCs, and remained active after exposure to IR. In addition, Jagged1 induced Bcl2 activation and p63 inhibition, which facilitated prevention of apoptosis by IR. In contrast, Notch2 depletion significantly reduced the radio-protective effect of MSC on HPSCs through down-regulation of Bcl2. This result implies that activated Jagged1-Notch2 interaction could decrease IR-induced p63 expression, thus limiting DNA damage in the hematopoietic system. A previous study showed that silencing Jagged1 inhibited cell growth and proliferation *in vitro* and *in vivo*^58^. Our *in vivo* data showed that MSC administration ameliorated radiation-induced BM failure and improved survival in mice, but Jagged1 knockdown of MSC was not able to prevent BM suppression and mortality (Fig. 6). Taken together, among Notch ligands, Jagged1 plays an important role in blocking radiation-induced damage of the hematopoietic system.

In conclusion, activated Jagged1 expression on MSCs enhances Notch2 signaling, which may prevent apoptosis of HSCs *in vitro* and improve hematopoietic recovery and reduce mortality *in vivo*. In addition, Notch2 reduces IR-induced p63 expression, leading to up-regulation of anti-apoptotic Bcl2 protein expression. We suggest that the activation of Notch by MSCs could be a successful strategy to treat patients with radiation exposure.

## Electronic supplementary material

Dataset1
