# Supplementary material for: Mesenchymal stem cell-mediated Notch2 activation overcomes radiation-induced injury of the hematopoietic system
Source: Sci Rep. 2018 Jun 18;8:9277. doi: 10.1038/s41598-018-27666-w (PMC6006282; doi:10.1038/s41598-018-27666-w)
Supplement: Supplementary file 1 — Dataset1 [file 41598_2018_27666_MOESM1_ESM.docx]

**Supplementary materials**

**Mesenchymal stem cell-mediated Notch2 activation overcomes radiation-induced injury of the hematopoietic system**

Areumnuri Kim^1^, Sehwan Shim^1^, Min-Jung Kim^1^, Jae Kyung Myung^1,2^, Sunhoo Park^1, 2^

^1^Laboratory of Radiation Exposure & Therapeutics, National Radiation Emergency Medical Center, ^2^Department of Pathology, Korea Institute of Radiological & Medical Science, Seoul, Korea

**Corresponding Author**: Sunhoo Park, M.D., Ph.D.

Department of Pathology, Korea Institute of Radiological & Medical Science

215-4 Gongneung-dong, Nowon-ku, 139-706, Seoul, Korea

(Phone) +82-2-3399-5803

(Fax) +82-2-970-1952

(E-mail) [sunhoo@kirams.re.kr](mailto:sunhoo@kirams.re.kr)

**Supplementary Table 1. Primers used for the Notch signaling and cytotoxicity.**

**
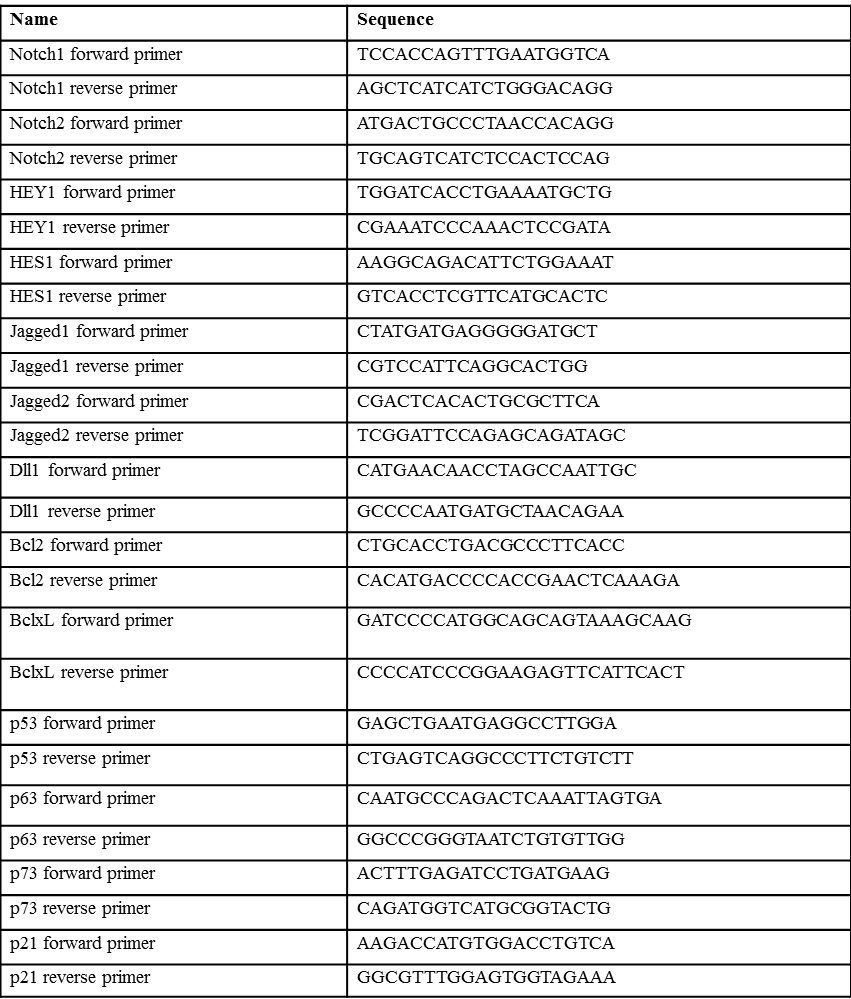
**

**
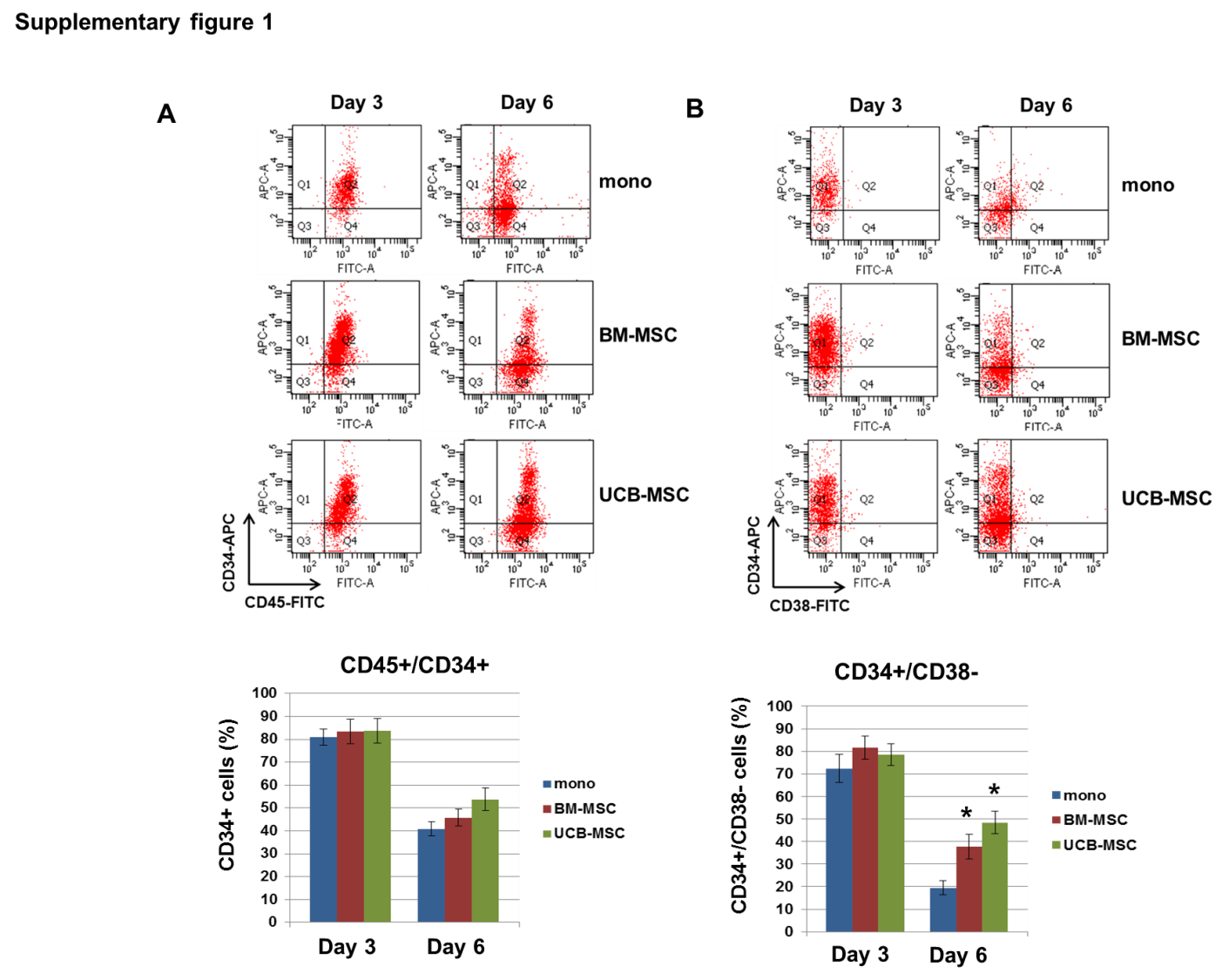
**

**Supplementary Figure 1.** HPSCs were co-cultured with UCB-or BM-MSCs. At days 3 and 6 after co-culture, CD45+CD34+ cells (A) and CD34+CD38- cells (B) were measured by flow cytometry.

**
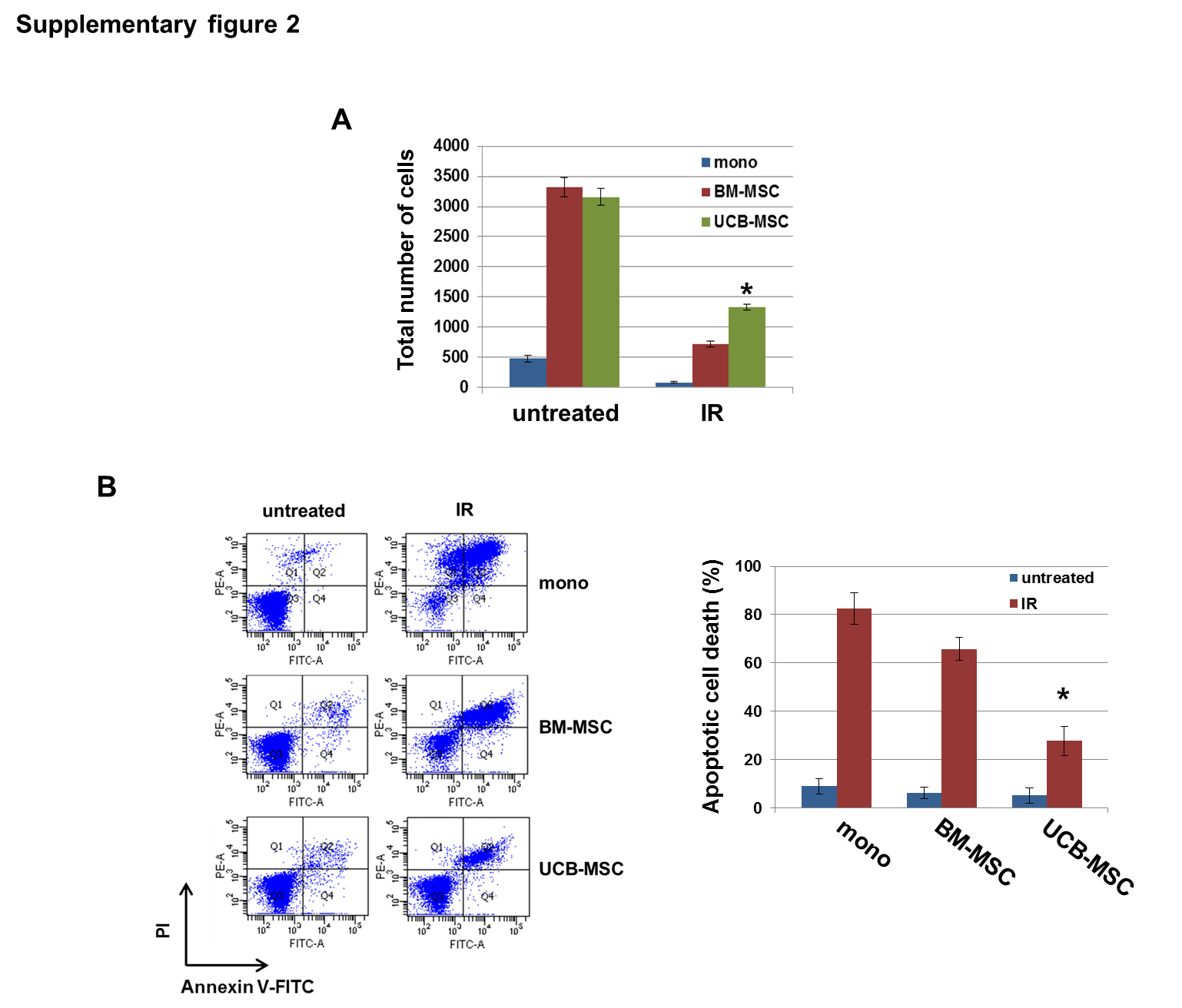
**

**Supplementary Figure 2.** HPSCs were exposed to IR (4 Gy) and then co-cultured with UCB-or BM-MSCs. Six days after IR, all hematopoietic cells were counted (A) and apoptotic cells (Annexin V+ and PI+) were measured by flow cytometry.

**
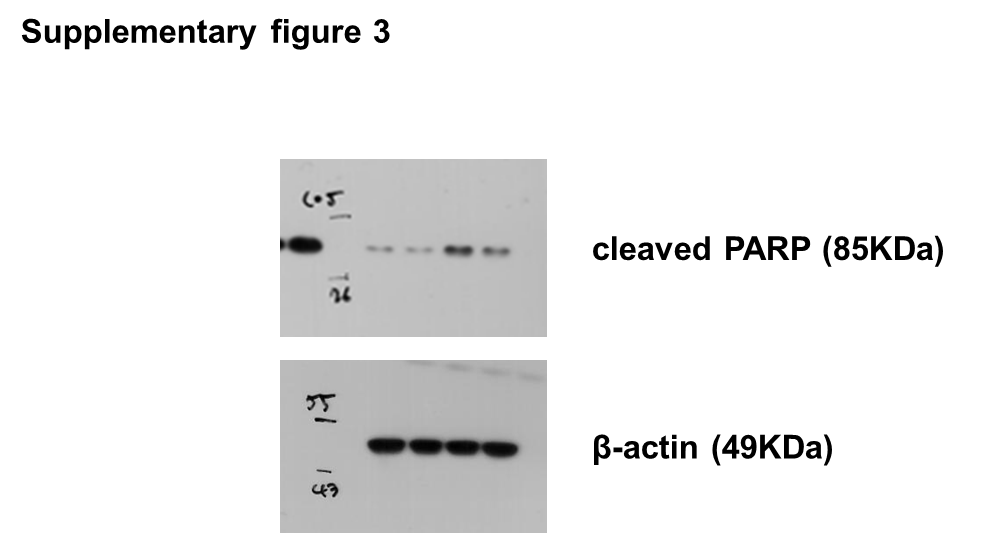
**

**3.** The blots corresponding to figure 2C.

**
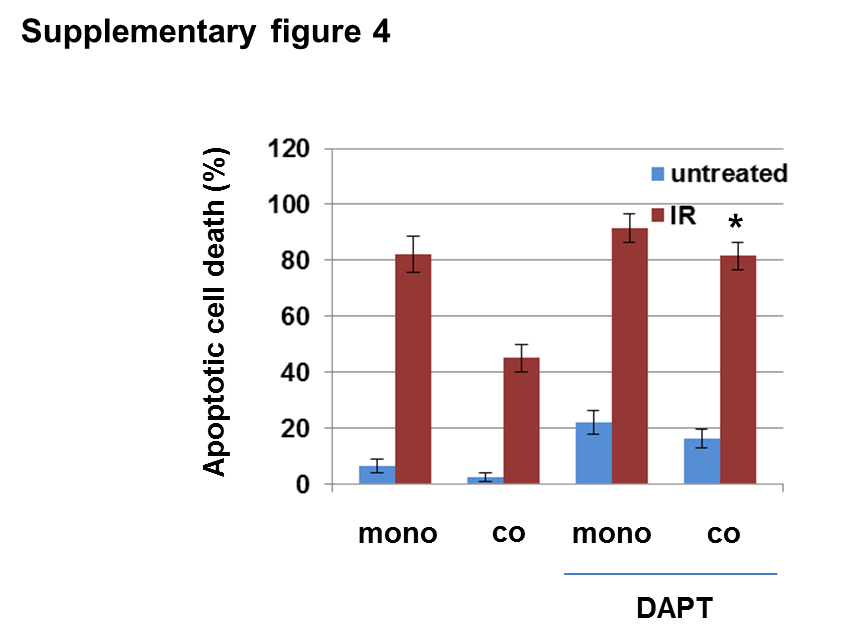
**

**Supplementary Figure 4.** Mono- or co-cultured HPSCs were treated with DAPT (10 μM), which was followed by IR (4 Gy). Three days after IR, cells were stained with Annexin V and propidium iodide (PI) to detect apoptotic cells by flow cytometry. *p<0.05, for mono-culture and co-culture


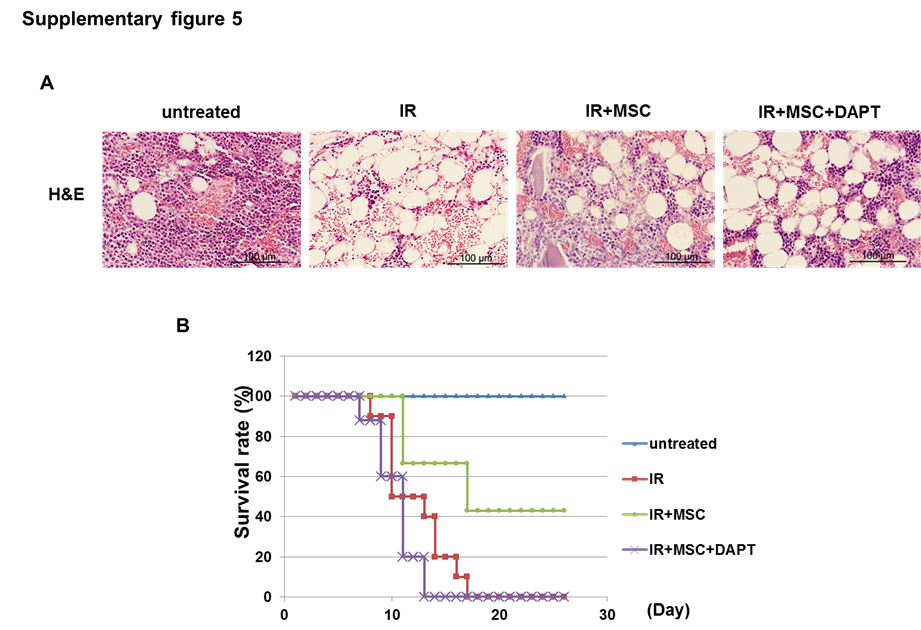


**Supplementary Figure 5.** Mice were exposed to whole body IR (6.5 Gy) followed by tail vein injection of MSCs and intra-peritoneal treatment with DAPT. (A) Representative images are shown for H&E staining of mouse femurs at 14 days after IR. Scale bar = 100 μm (B) Mice were observed for survival over 25 days (n = 10). The graph shows survival curves.

**
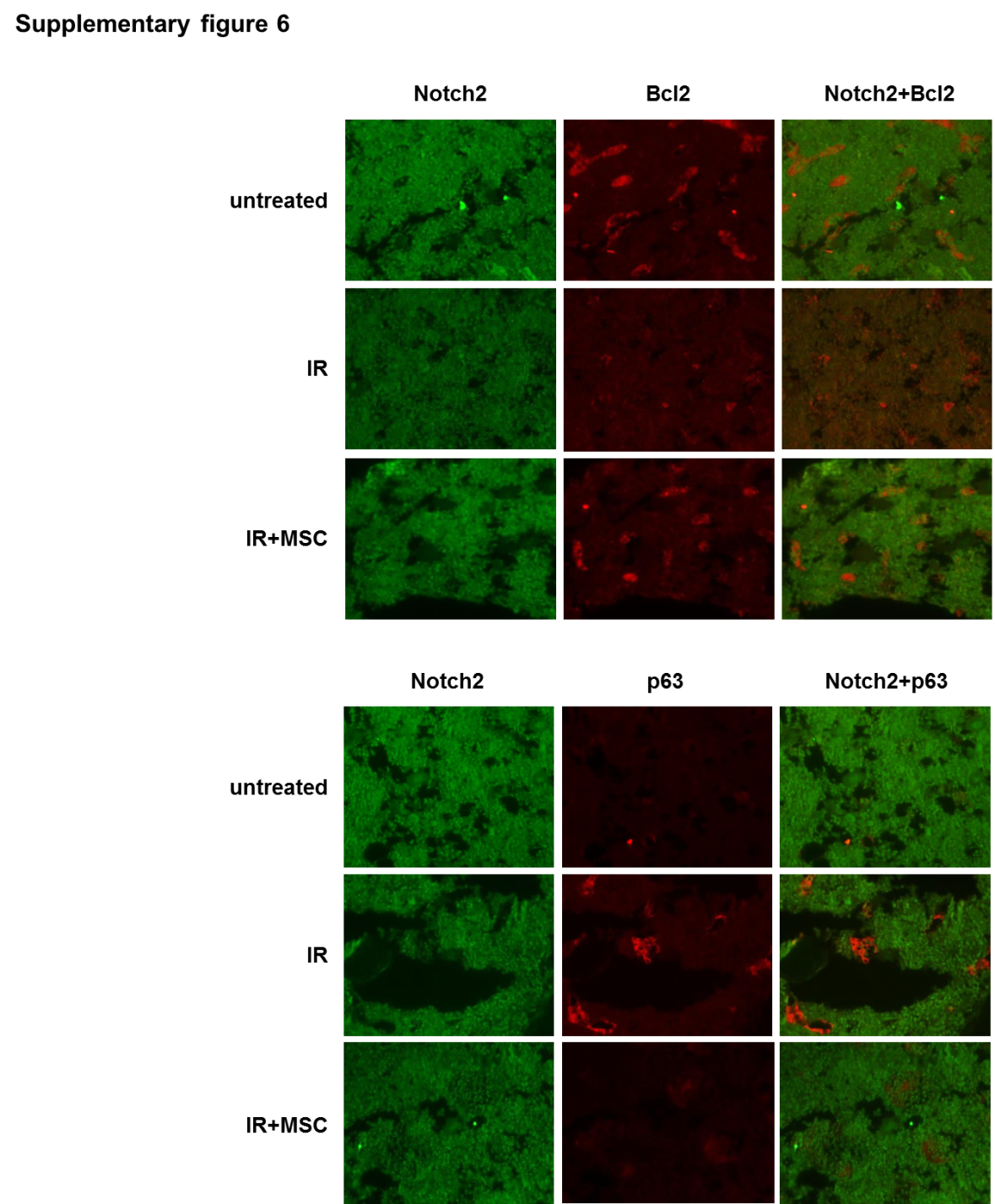
**

**Supplementary Figure 6.** Mice were exposed to whole body IR (6Gy) followed by tail vein injection of MSCs (1 × 10^6^ cells/mouse). Two weeks after IR, Notch2, p63, and Bcl2 expression were detected in mouse BM using immunofluorescence staining. ×400. Representative images are shown for H&E staining of mouse femurs at 14 days after IR.
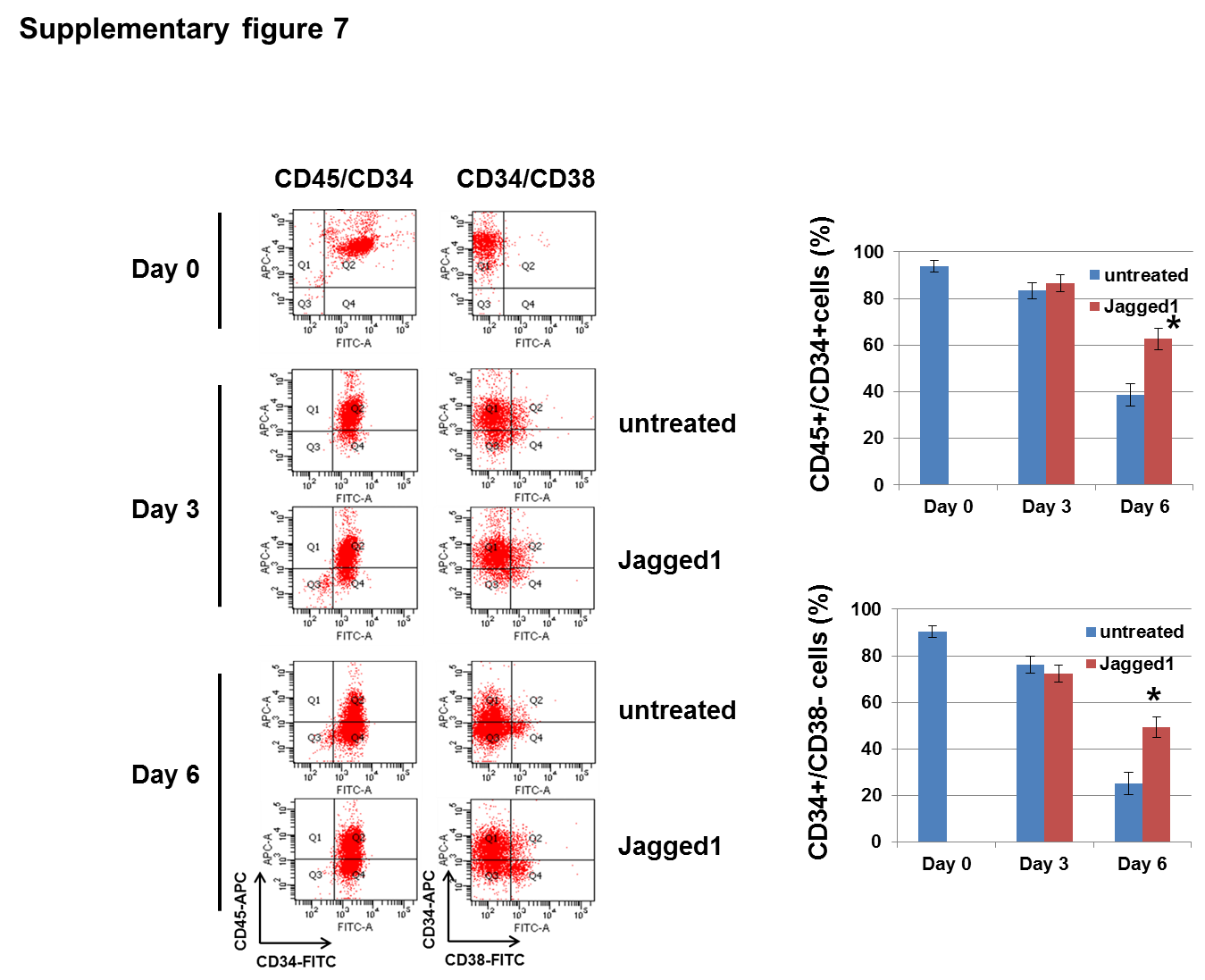


**Supplementary Figure 7.** HPSCs showed >90% CD34+CD38- cells at Day 0. HPSCs were treated with or without Jagged1 (1 μg/mL) for 3 and 6 days. Expression of CD45, CD34, and CD38 was analyzed by flow cytometry. *p<0.05, for untreated and Jagged1-treated.

**
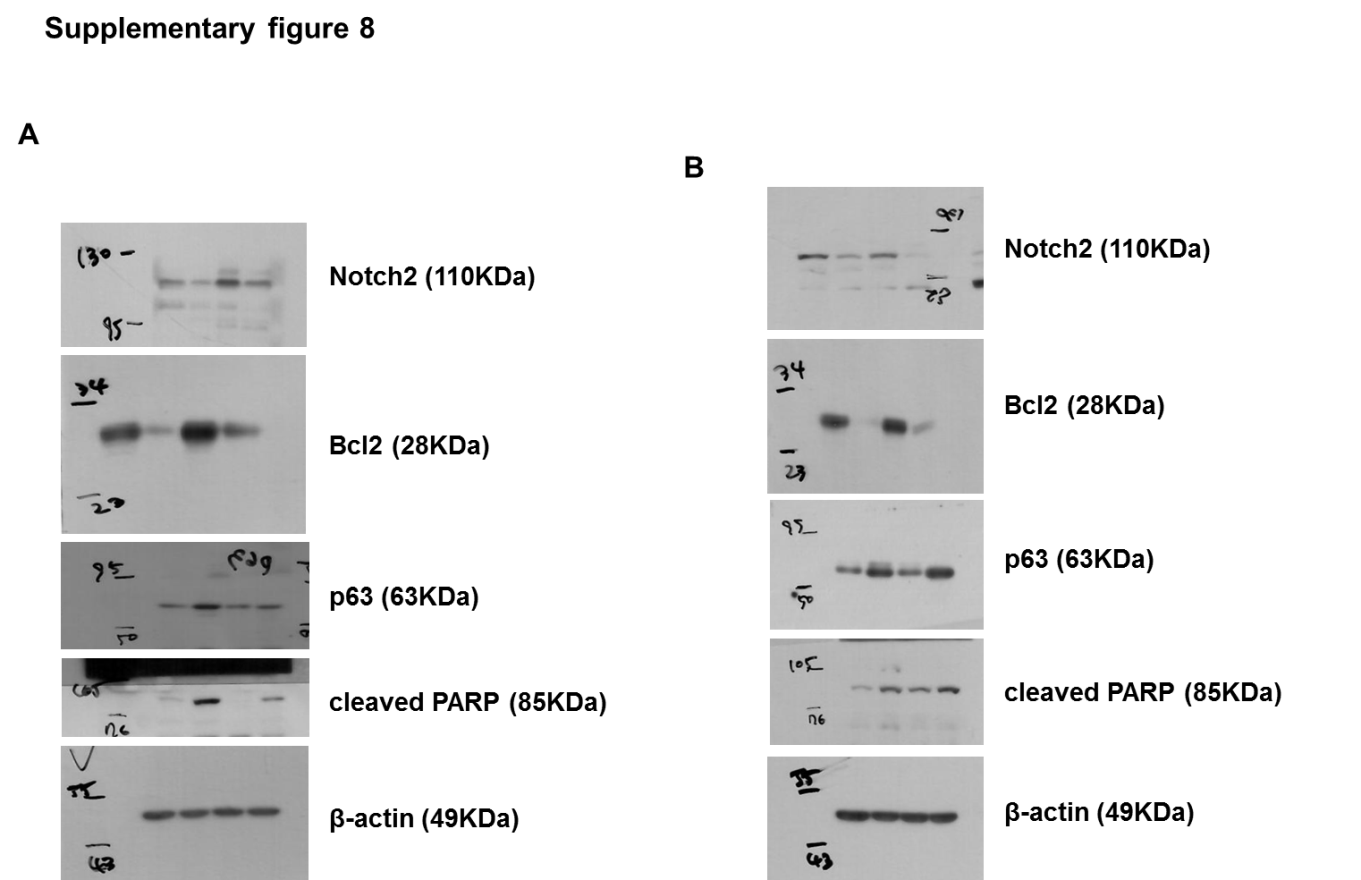
**

**Supplementary Figure 8.** (A) The blots corresponding to figure 5A (A) and figure 5C (B).


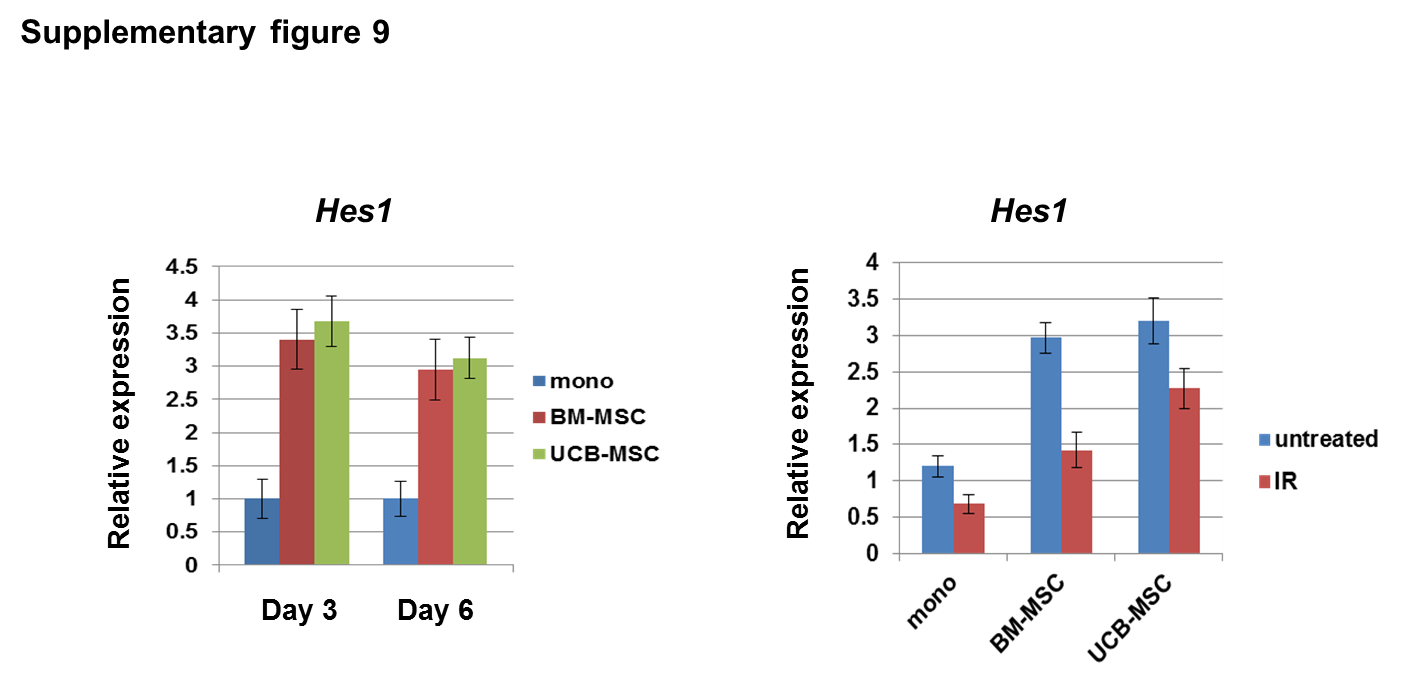


**Supplementary Figure 9.** HPSCs were co-cultured with BM-MSCs or UCB-MSCs. At day 3 and 6 after co-culture, Hes1 level was analyzed in HPSCs using qPCR (left panel). HPSCs were subjected to IR (4 Gy) and cultured with BM-MSCs or UCB-MSCs. At day 3 post-IR, the expression levels of Hes1 was analyzed in HPSCs using qPCR (right panel).


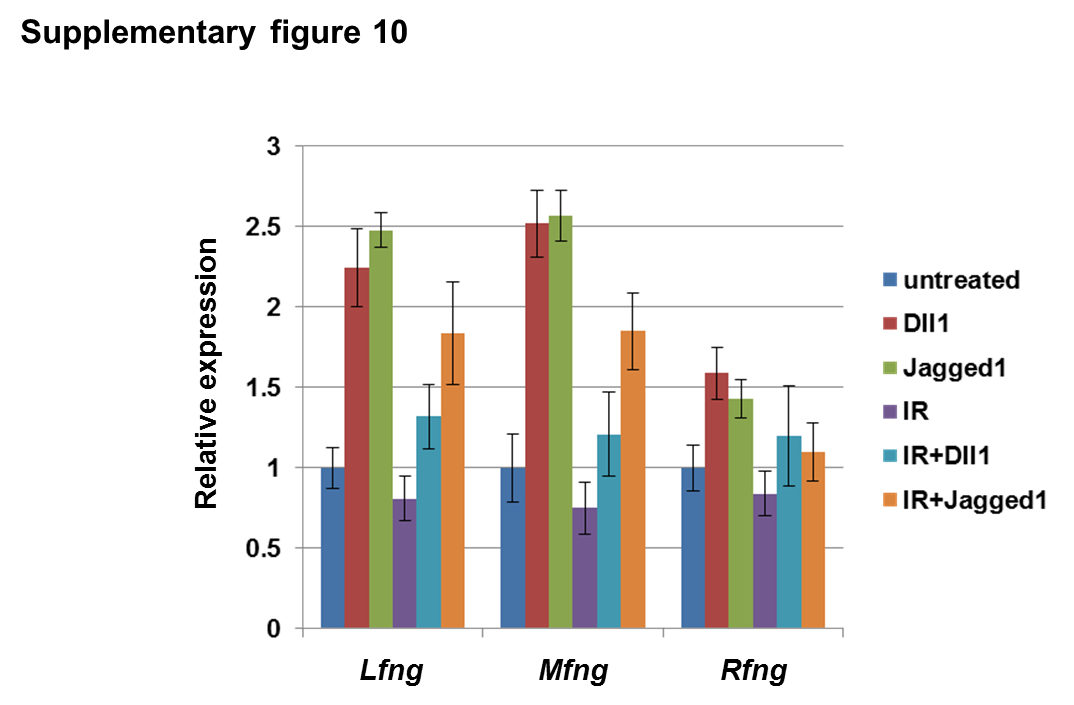


**Supplementary Figure 10.** HPSCs were exposed to IR (4 Gy) and then treated with Jagged1 or Dll1 for 3 days. The expression of Lfng, Mfng and Rfng were measured by qPCR.
